# Neutrophils are shaped by the tumor microenvironment: novel possibilities for targeting neutrophils in cancer

**DOI:** 10.1038/s41392-024-01786-4

**Published:** 2024-04-02

**Authors:** Carlos Silvestre-Roig, Lydia Kalafati, Triantafyllos Chavakis

**Affiliations:** 1 Institute of Experimental Pathology (ExPat), Center for Molecular Biology of Inflammation (ZMBE), University of Münster, Münster, Germany; 2 Institute for Clinical Chemistry and Laboratory Medicine, University Hospital and Faculty of Medicine, Technische Universität Dresden, Dresden, Germany

**Keywords:** Tumour immunology, Innate immune cells

In a recent paper published in *Science*, Ng et al. provide seminal insights into the process through which neutrophils are reprogrammed by the tumor environment, leading to a convergent transition from different neutrophil states towards a unique end-stage differentiated pro-tumor phenotype.^1^ These findings have major translational implications as they provide potential neutrophil-related targets for tumor immunotherapy.

Neutrophils are increasingly recognized as effector cells with phenotypic and functional plasticity. Their ability to adapt to their surrounding environment is exemplified by organ-specific changes in phenotype and function,^2^ a process that occurs in a short period of time, tailored to their limited lifespan. Emerging evidence indicates that neutrophils display substantial phenotypic and functional heterogeneity within the tumor microenvironment as well.^3^ Neutrophils are a significant fraction of the immune cells infiltrating tumors, and their numbers may be often underestimated.^4^ Tumor-associated neutrophils (TANs) may display pro-tumor actions, for instance, by promoting angiogenesis or immunosuppression, and anti-tumor actions, in parts due to tumoricidal properties.^3–5^ Nevertheless, the accumulation of TANs usually correlates with an adverse prognosis in most human cancers, and/or an impaired response to anticancer therapy.^3,4^ Hence, the default scenario is that tumors hijack neutrophils towards a tumor-promoting phenotype, although the underlying mechanisms are poorly understood. In particular, a gap in knowledge exists with regard to the anatomical site where neutrophils are primed towards acquiring a pro-tumoral state, as well as the specific contribution of ontogeny and tissue-derived signals to this process.

The elegant study by Ng et al. integrated analysis at single cell level, specifically, RNA sequencing (scRNAseq) and assay for transposase-accessible chromatin using sequencing (ATACseq) to phenotypically study the heterogeneity of neutrophils from the bone marrow (BM), spleen, blood and tumor; in the latter case by employing a model of orthotopic pancreatic cancer.^1^ These analyses delineated the differentiation trajectories of neutrophils from their progenitor cells (in the BM and spleen) to the more mature cells in the circulation. Interestingly, the neutrophil subpopulations identified in the tumor (referred to as T1, T2, T3) clustered separately than neutrophils from other locations, indicating rewiring of their transcriptomic profile within the tumor microenvironment. No alternative differentiation pathways from progenitors to these neutrophils were observed in this study, thus, implying that these transcriptomic profiles of TANs are likely acquired after infiltration to the tumor site.^1^

Transcriptionally, the T3 neutrophil subpopulation exhibited a pro-tumorigenic phenotype characterized by increased glycolysis, hypoxia-signaling and an angiogenesis-promoting profile. This T3 neutrophil signature was largely conserved across different tumor types, as assessed in other published scRNAseq datasets. ATACseq analysis revealed that the T3 signature was linked to epigenetic reprogramming of neutrophils at the tumor site.^1^ To accurately define these subpopulations phenotypically, the authors used a multiplexed surface protein screen of 249 surface markers and identified a set of immunomodulatory and suppressive markers associated with the T3 neutrophil subset, including dcTRAIL-R1, PD-L1, and CD371, amongst others. The receptor dcTRAIL-R1 was mainly expressed by TANs of not only pancreatic but also other mouse tumors, including breast and lung cancer.^1^ Hence, dcTRAIL-R1 stands out as an important marker for the identification of the pro-tumor T3 neutrophil phenotype by flow cytometry or tissue imaging techniques in mice. A human homolog for this marker remains to be identified.

**Fig. 1:**
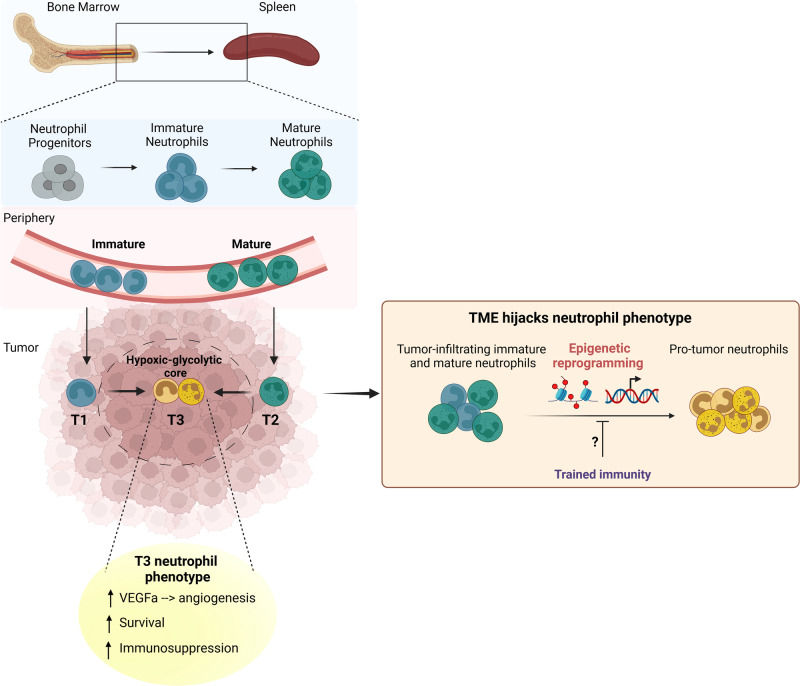
Neutrophil hijacking by tumors. BM- and spleen-derived neutrophils of immature and mature stages that are discriminated by the presence of CD101 enter the tumor site and undergo epigenetic and transcriptomic reprogramming in the local tumor microenvironment (TME). In tumors, immature T1 (dcTRAIL-R1-negative CD101-negative) and mature T2 (dcTRAIL-R1-negative CD101-positive) cells give rise to a terminally differentiated T3 neutrophil population with intermediate maturation status, characterized by expression of dcTRAIL-R1, prolonged survival and pro-tumor functions, including immunosuppressive activities. T3 neutrophils are localized in the hypoxic-glycolytic core of the tumor and display high expression of vascular endothelial growth factor alpha (VEGFα), hence facilitating cancer angiogenesis and growth. Induction of trained immunity could potentially abrogate this reprogramming of neutrophils by the tumor environment; this hypothesis should be interrogated in future studies. The figure is inspired by and drawn based on the article by Ng et al.^1^ Created with Biorender.com

Given that neutrophils of different maturation states coexist within the tumor environment, the authors also interrogated the maturation level of the identified T1–T3 neutrophil subpopulations. Based on annotated maturation gene signatures, T1 represented a more immature neutrophil population, T2 were mature neutrophils, while T3 represented a mixed population with different degrees of maturation. To study the differentiation of these subpopulations within the tumor tissue, the authors used trajectory analysis algorithms that predicted differentiation from both T1 and T2 to T3 (Fig. 1). Consistent with this prediction, adoptively transferred T1 (dcTRAIL-R1-negative, CD101-negative) and T2 (dcTRAIL-R1-negative, CD101-positive) cells were converted to dcTRAIL-R1-positive T3 neutrophils. Similar results were obtained when T1 or T2 neutrophils were incubated with tumor-conditioned media. These data suggest a tumor-specific imprinting of neutrophils, akin to their irreversible differentiation to the T3 phenotype, irrespective of their original maturation status.^1^

The tumor microenvironment is characterized by heterogeneity, displaying a regional distribution of areas enriched in stromal cells (in the periphery) and necrotic areas characterized by elevated hypoxia at the core of the tumor. To better understand how local cues regulate the tumor-driven neutrophil adaptation and the emergence of the aforementioned neutrophil states, the authors combined spatial transcriptomics and multiplexed tissue imaging techniques to identify the T1–T3 subsets by using their distinct transcriptional signatures in combination with dcTRAIL-R1 expression. Interestingly, T3 neutrophils with a glycolytic and hypoxic transcriptomic profile were located at the tumor core, while T1 as well as T2 cells remained in the periphery. Within the core of the tumor, T3 neutrophils were enriched in highly hypoxic and glycolytic areas (Fig. 1), supporting their specific adaptation that enables them to survive in this environment.^1^ Interestingly, in vitro assays using tumor-conditioned media under hypoxia showed that neutrophils acquired the T3 phenotype independently of oxygen levels. Functionally, and in line with their pro-angiogenic profile at the transcriptomic level, T3 neutrophils exhibited an enhanced capacity to promote vascular growth and tumor development (Fig. 1), as demonstrated by the enhanced tumor growth upon transfer of T3 neutrophils concomitantly with tumor cell injection.^1^ Notably, therapeutic blockade of dcTRAIL-R1 with antibodies prevented the pro-tumorigenic activities of T3 neutrophils. Importantly, the transcriptional programs from T1 to T3 neutrophils were identified in human pancreatic cancer datasets and used to predict patient survival. Specifically, the T3 neutrophil profile showed a strong association with low patient survival, as compared to T1 and T2 phenotypes, highlighting the potential prognostic value of TAN signatures.^1^

In conclusion, this manuscript demonstrates how neutrophils are hijacked by the tumor microenvironment to tailor their functions toward promoting tumor growth. These findings have many important implications, for instance, they provide neutrophil-related targets for novel cancer immunotherapies. An open question is whether it is feasible to interfere with this tumor-mediated reprogramming of neutrophils and thereby prevent the emergence of the pro-tumoral T3 neutrophil state; this should be investigated in preclinical tumor models in the future. In this context, trained immunity, representing innate immune memory, has been shown to promote anti-tumor activities via rewiring of granulopoiesis and neutrophils.^5^ Future studies should therefore also address if induction of trained immunity could counteract the hijacking of neutrophils by tumors.
